# Time course of neuropathological events in hyperhomocysteinemic amyloid depositing mice reveals early neuroinflammatory changes that precede amyloid changes and cerebrovascular events

**DOI:** 10.1186/s12974-019-1685-z

**Published:** 2019-12-30

**Authors:** Erica M. Weekman, Tiffany L. Sudduth, Brittani R. Price, Abigail E. Woolums, Danielle Hawthorne, Charles E. Seaks, Donna M. Wilcock

**Affiliations:** Department of Physiology, Sanders-Brown Center on Aging, University of Kentucky, 800 S. Limestone St., Lexington, KY 40536 USA

**Keywords:** Alzheimer’s disease, Vascular contributions to cognitive impairment and dementia, Hyperhomocysteinemia, Neuroinflammation, Astrocytic end-feet, Microhemorrhage

## Abstract

**Background:**

Vascular contributions to cognitive impairment and dementia (VCID) are the second leading cause of dementia behind only Alzheimer’s disease (AD); however, VCID is commonly found as a co-morbidity with sporadic AD. We have previously established a mouse model of VCID by inducing hyperhomocysteinemia in both wild-type and amyloid depositing mice. While we have shown the time course of neuropathological events in the wild-type mice with hyperhomocysteinemia, the effect of amyloid deposition on this time course remains unknown; therefore, in this study, we determined the time course of neuropathological changes in our mouse model of hyperhomocysteinemia-induced VCID in amyloid depositing mice.

**Methods:**

APP/PS1 mice were placed on either a diet deficient in folate and vitamins B6 and B12 and enriched in methionine to induce hyperhomocysteinemia or a control diet for 2, 6, 10, 14, or 18 weeks. Immunohistochemistry and gene expression analysis were used to determine neuroinflammatory changes. Microhemorrhages and amyloid deposition were analyzed using histology and, finally, behavior was assessed using the 2-day radial arm water maze.

**Results:**

Neuroinflammation, specifically a pro-inflammatory phenotype, was the first pathological change to occur. Specifically, we see a significant increase in gene expression of tumor necrosis factor alpha, interleukin 1 beta, interleukin 6, and interleukin 12a by 6 weeks. This was followed by cognitive deficits starting at 10 weeks. Finally, there is a significant increase in the number of microhemorrhages at 14 weeks on diet as well as redistribution of amyloid from the parenchyma to the vasculature.

**Conclusions:**

The time course of these pathologies points to neuroinflammation as the initial, key player in homocysteine-induced VCID co-morbid with amyloid deposition and provides a possible therapeutic target and time points.

## Background

Vascular contributions to cognitive impairment and dementia (VCID) are increasingly recognized as a significant cause of dementia, behind only Alzheimer’s disease (AD) [1]. Furthermore, VCID co-morbid with AD is extremely common, estimated to occur in at least 60% of AD cases [2]. Clinical-neuropathological correlation data has revealed that cerebrovascular pathologies increase the likelihood of dementia for a given amount of AD pathology. In early-onset familial AD, cerebrovascular abnormalities as detected by neuroimaging appear to precede detectable AD pathology. Despite our increasing understanding of VCID as a contributing factor to clinical dementia, our appreciation for the mechanistic underpinnings of VCID, and also identification of potential therapeutic targets to treat VCID, has been lacking, in part due to a lack of relevant animal models.

One of the challenges of developing models for VCID is that VCID is an umbrella term for a variety of cerebrovascular pathologies including micro- and macro-infarcts, micro- and macro-hemorrhages, cerebral hypoperfusion, periventricular and deep white matter hyperintensities, and stroke. We have been working on a mouse model of VCID characterized by inflammation, microhemorrhages, and cerebral hypoperfusion. The model is induced through diet modification to elevate plasma homocysteine, a non-protein forming amino acid (Hcy), resulting in hyperhomocysteinemia (HHcy). Over 20 years ago, HHcy (elevated plasma homocysteine) was identified as an independent risk factor for stroke and vascular disease [3]. HHcy is associated with confirmed VCID and AD cases [4] and is recognized as an important risk factor for AD [5]. HHcy is associated with accelerated hippocampal atrophy [4] and cognitive loss in AD patients [6]. The association with hippocampal atrophy appears to be independent of Aβ pathology [7]. HHcy [8–11] and/or low B12 [12] and folate levels [13, 14] are also accompanied by white matter lesions, indicative of vascular damage. Genetic mutations in cystathionine β synthase, a key enzyme in the homocysteine metabolism pathway, lead to HHcy and are recognized as a cause of stroke in children and young adults [15].

We have previously shown that induction of HHcy in wild-type (WT) mice leads to neuroinflammation, microhemorrhages., and cognitive impairment, producing a clinically relevant mouse model of VCID [16]. We have also shown that HHcy leads to astrocytic end foot disruption beginning between 6 and 10 weeks on diet [17]. In our co-morbidity model, APP/PS1 mice placed on the HHcy diet show a switch to a pro-inflammatory phenotype, microhemorrhages, additive cognitive deficits, and a redistribution of amyloid from the parenchyma to the vasculature [18]. While we have previously described the time course of neuropathological changes in our WT mice on the HHcy diet, the effect of amyloid deposition on this time course remains unknown. In this study, we examined neuroinflammation, microhemorrhages, amyloid deposition, and cognition along a time course of 2, 6, 10, 14, and 18 weeks in our co-morbidity model. Similar to our WT HHcy model, neuroinflammation appears first at 6 weeks, followed by significant cognitive deficits at 10 weeks, and finally, microhemorrhages and redistribution of amyloid from the parenchyma to the vasculature occurred after 14 weeks on diet.

## Materials and methods

### Animals

Female and male APP/PS1 mice (C57BL/6 mice carrying human APPSwe and PS1-dE9 mutations) were bred in house and aged 6–8 months before starting a diet deficient in folate and vitamins B6 and B12 and enriched in methionine (Envigo TD130867; Envigo, Indianapolis, Indiana) or a control diet with normal levels of folate, vitamins B6 and B12, and methionine (Envigo TD01636; Envigo, Indianapolis, Indiana). Mice were on diet for 2, 6, 10, 14, or 18 weeks. Sample sizes, sex distribution, and homocysteine levels are summarized in Table 1. Homocysteine levels were measured by the clinical laboratory at the University of Kentucky Clinic. At each time point, the APP/PS1 mice on the HHcy diet had significantly higher levels of homocysteine compared to mice on the control diet. This study was approved by the University of Kentucky Institutional Animal Care and Use Committee and conformed to the National Institutes of Health Guide for the Care and Use of Animals in Research.

**Table 1:** Sample size, sex distributions, and homocysteine levels

Weeks on diet (HHcy or Cont diet)	APP/PS1			
	F	Mean plasma Hcy (μmol/L)	M	Mean plasma Hcy (μmol/L)
2—HHcy	3	42.2 ± 12.1	2	39.5 ± 10.1
2—Cont	3	6.2 ± 0.8	3	4.9 ± 1.8
6—HHcy	5	64.4 ± 8.2	5	59.3 ± 9.8
6—Cont	6	5.2 ± 0.9	6	5.5 ± 0.8
10—HHcy	4	69.3 ± 6.2	3	71.6 ± 5.1
10—Cont	6	4.4 ± 1.8	6	6.6 ± 1.2
14—HHcy	5	88.6 ± 14.1	5	77.2 ± 6.9
14—Cont	7	5.6 ± 1.4	7	4.8 ± 1.1
18—HHcy	2	71.5 ± 7.8	5	64.3 ± 8.4
18—Cont	6	4.9 ± 0.7	6	5.7 ± 0.5

### Behavior testing

The 2-day radial arm water maze behavior test was performed the week prior to tissue harvesting at the University of Kentucky Rodent Behavior Core. The 20day behavior task was performed as previously described [19]. Briefly, a 6-arm maze was submerged in a pool of water with a goal platform placed at the end of one arm. Each mouse performed 15 trials over the 2 days and began each trial in a different arm while the goal platform remained the same. The number of errors (incorrect arm entries) was counted over a 60-s period. Errors were averaged for three trials resulting in 10 blocks over the 2 days. Blocks 1–5 comprised day 1 trials while blocks 6–10 comprise day 2 trials.

### MRI

Mice were imaged by T2* MRI the week prior to tissue harvesting. Mice were imaged with a 7-T Bruker ClinScan MRI system (Bruker, Billerica, MA) with an MRI CryoProbe, delivering 2.5 times the signal to noise of a standard room temperature radiofrequency coil, located at the Magnetic Resonance Imaging and Spectroscopy Center at the University of Kentucky. Fourteen coronal slices were acquired with a FLASH sequence with a TR 165 ms, TE 15.3 ms, 25° flip angle, 448 × 448 matrix, 0.4 mm thick, 20% gap, 0.033 mm × 0.033 mm resolution, 10 averages, and TA 24 min. Mice were anesthetized with 1.3% isoflurane using an MRI compatible vaporizer. They were then positioned prone and held in place on the scanning bed using tooth and ear bars. The animals were maintained at 37° with a water-heated scanning bed. Body temperature, heart, and respiration rates were monitored. T2* MRI images were analyzed by one blinded investigator who identified abnormalities that resembled hemorrhagic infarcts. These infarcts were counted, and this number was normalized to the number of images counted to provide a per section count.

### Tissue processing and histology

After a lethal injection of Beuthanasia-D, blood was collected for plasma and the mice were perfused with 25 mL normal saline. Brains were rapidly removed and bisected along the mid-sagittal plane. The left half was immersion fixed in 4% paraformaldehyde for 24 h while the right half was dissected into the frontal cortex, posterior cortex, hippocampus, and the rest of brain and flash frozen in liquid nitrogen and then stored at − 80 °C. The left hemibrain was passed through 10%, 20%, and finally 30% sucrose solutions for cryoprotection prior to sectioning. Using a sliding microtome, 25-μm frozen horizontal sections were collected and stored free floating in 1× DPBS-containing sodium azide at 4 °C.

Eight sections (25 μm thick) spaced 600 μm apart were selected for free floating immunohistochemistry for CD11b (rat monoclonal, 1:1000 dilution, AbD Serotec, Raleigh, NC for the 6–14-week time points; rat monoclonal, 1:1000 dilution, BioLegend, San Diego, CA, for the 18-week time point). To reduce variability due to the use of two separate CD11b antibodies, we analyzed the data as a percent of the control mice for that specific time point (with the control mice averaging 100%) rather than direct comparison of the percent areas occupied by positive immunostain. Immunohistochemistry was performed as previously described [20]. Stained sections were mounted, air dried overnight, dehydrated, and coverslipped in DPX (Electron Microscopy Sciences, Hatfield, PA). Analysis was performed by measuring the percent area occupied by positive immunostain using the Nikon Elements AR image analysis system (Nikon Instruments, Melville, NY) as described previously [21].

Eight sections spaced 600 μm apart were selected and mounted on slides for Congo red and Prussian blue staining as previously described [22, 23]. Congo red analysis was performed using the Zeiss Axio Scan.Z1 Slide Scanner (Carl Zeiss Microscopy, Jena, Germany) and the Nikon Elements AR image analysis system. Neutral red was used as a counterstain for Prussian blue. For analysis of microhemorrhages, Prussian blue-positive profiles across the entire hemibrain section were counted by a blinded individual and calculated as an average per section.

### Aβ ELISA

Biochemical analysis of Aβ levels was performed as previously described [18]. Briefly, soluble protein was extracted from the right frontal cortex using PBS with complete protease and phosphatase inhibitor (Pierce Biotechnology). After centrifugation, the supernatant was labeled the “soluble” extract, and the pellet was homogenized in 70% formic acid. After centrifugation and neutralization with 1 M Tris-HCl, the supernatant was labeled the “insoluble” extract. Protein concentrations were determined using the BCA protein assay kit (Pierce Biotechnology). The Meso-Scale Discovery multiplex ELISA system was used to measure Aβ1–38, Aβ1–40, and Aβ1–42 levels in the soluble and insoluble extracts (V-PLEX Aβ Peptide Panel 1 (6E10) Kit; MSD).

### Quantitative real-time RT-PCR

RNA was extracted from the right hippocampus using the E.Z.N.A. total RNA kit (Omega-Bio-Tek, Norcross, GA) according to the manufacturer’s instructions. RNA was quantified using the Biospec nano spectrophotometer (Shimadzu, Japan) and reverse transcribed to cDNA using the cDNA High Capacity kit (Thermo Fisher, Grand Island, NY) according to the manufacturer’s instructions. RT-PCR was performed using the Fast TaqMan Gene Expression assay (Thermo Fisher). In each well of a 96-well plate, 0.5 μL cDNA (100 ng, based on RNA concentrations) was diluted with 6.5 μL RNase-free water. One microliter of the appropriate gene probe was added with 10 μL of Fast TaqMan to each well. Target amplification was performed using the ViiA7 (Applied Biosystems, Grand Island, NY). All genes were normalized to 18S rRNA and the fold change was determined using the −ΔΔCt method [24]. Table 2 shows the genes tested along with their PMID and TaqMan ID.

**Table 2:** Genes for RT-PCR

Gene of interest	PMID	TaqMan ID
TNFα	NM_013693.3	Mm.1293
IL1β	NM_008361.3	Mm.222830
IL12a	NM_008351.2	Mm.103783
IL-6	NM_031168.1	Mm.1019
IL10	NM_010548.2	Mm.874
YM1	NM_009892.2	Mm.387173

### Data analysis

Data are presented as mean ± SEM. Statistical analysis was performed using the JMP statistical analysis software program (SAS Institute, Cary, NC). Radial arm water maze data were analyzed by repeated measures ANOVA. We also performed Student’s *t* test on individual block data. Histological, immunohistochemical, and biochemical data were analyzed by one-way ANOVA for each time point, as well as across time points. Statistical significance was assigned where the *P* value was lower than 0.05.

## Results

**Fig. 1:**
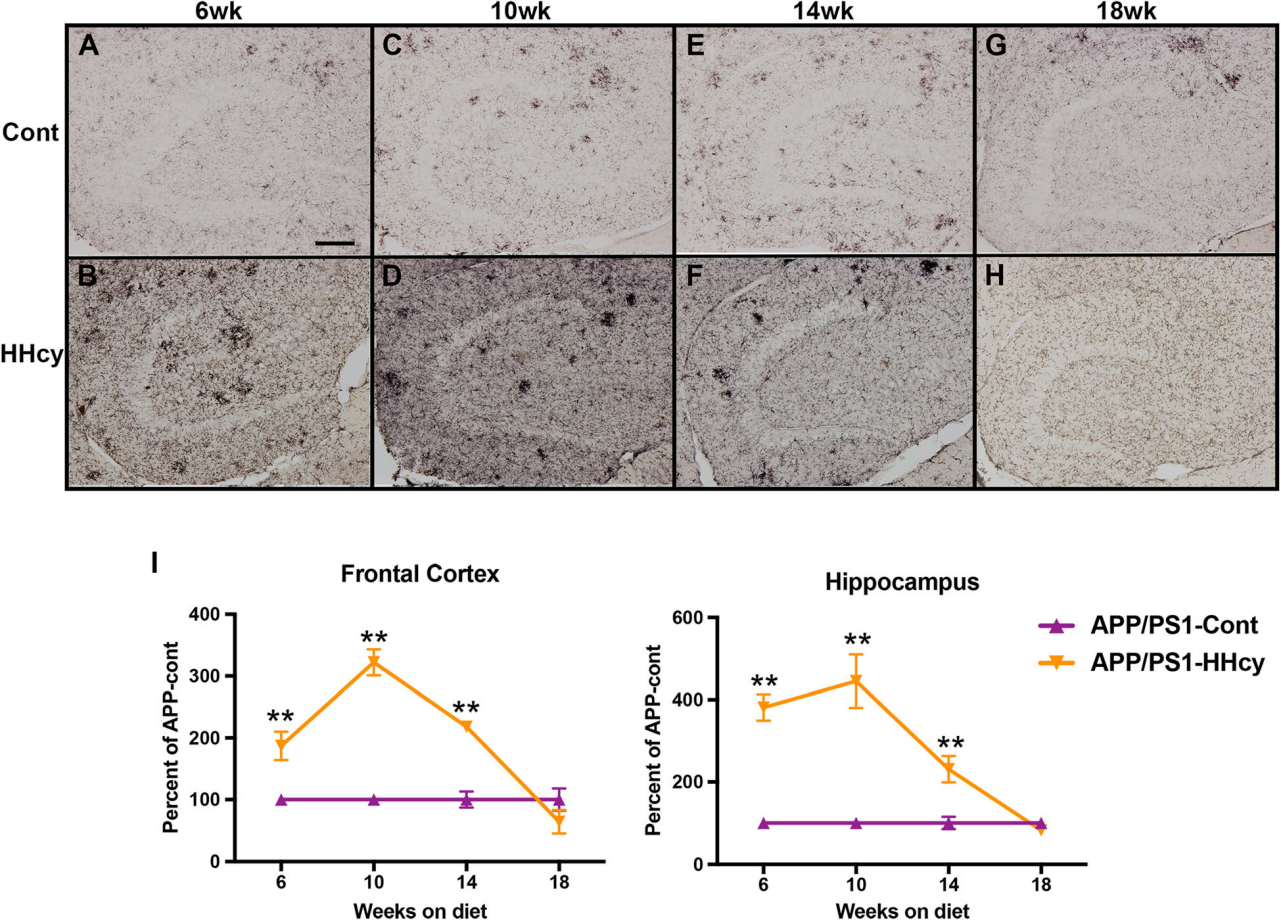
Neuroinflammatory changes begin at 6 weeks on the HHcy diet in the APP/PS1 mice. **a–h** Representative images of CD11b staining in the dentate gyrus of APP/PS1 mice on control diet or HHcy diet for 6, 10, 14, or 18 weeks are shown. Scale bar in *A* = 100 μm. **i** Quantification of percent positive stain in the frontal cortex and hippocampus

Neuroinflammation is the first pathological abnormality that we observe in our APP/PS1-HHcy model. Immunohistochemistry for CD11b, which stains for both activated and resting microglia, showed a similar trend as the WT mice on the HHcy diet (previously published [17]). By 6 weeks on diet, the APP/PS1 mice on the HHcy diet have significantly more CD11b staining compared to APP/PS1 mice on the control diet (Fig. 1a, b, i). This increase in staining is sustained at 10 and 14 weeks on the HHcy diet but is reduced to control levels by 18 weeks (Fig. 1c–i).

**Fig. 2:**
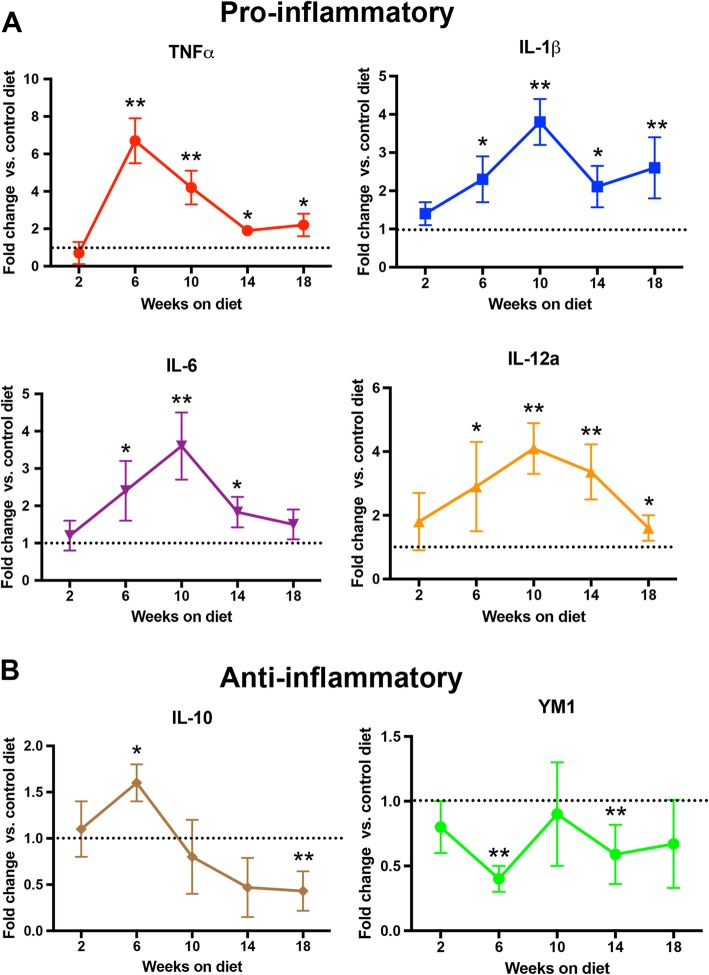
Pro-inflammatory markers are increased at 6 weeks on the HHcy diet. Relative gene expression for pro- (**a**) and anti- (**b**) inflammatory markers. Data are presented as fold change from APP/PS1 mice on control diet at that time point. * indicates *P* < 0.05, ** indicates *P* < 0.01 compared to APP/PS1 mice on control diet for that time point

While immunohistochemistry for CD11b showed an increase in microglial staining, we were interested in the specific cytokines expressed. The pro-inflammatory markers TNFα and IL-1β were significantly increased in the APP/PS1 mice on the HHcy diet starting at 6 weeks on diet and remained elevated through 18 weeks on diet (Fig. 2a). IL-12a and IL-6, anther two pro-inflammatory markers, were significantly elevated at 6, 10, and 14 weeks but returned to control levels by 18 weeks on the HHcy diet (Fig. 2a). The anti-inflammatory marker IL-10 was significantly elevated after 6 weeks on the HHcy diet but decreased by 10 weeks and was significantly decreased compared to controls by 18 weeks (Fig. 2b). Another anti-inflammatory marker, YM1, was significantly decreased at 6 and 14 weeks on the HHcy diet (Fig. 2b). Similar to the CD11b staining, the increases in pro-inflammatory cytokines starting at 6 weeks were also seen in the WT mice on the HHcy diet [17].

**Fig. 3:**
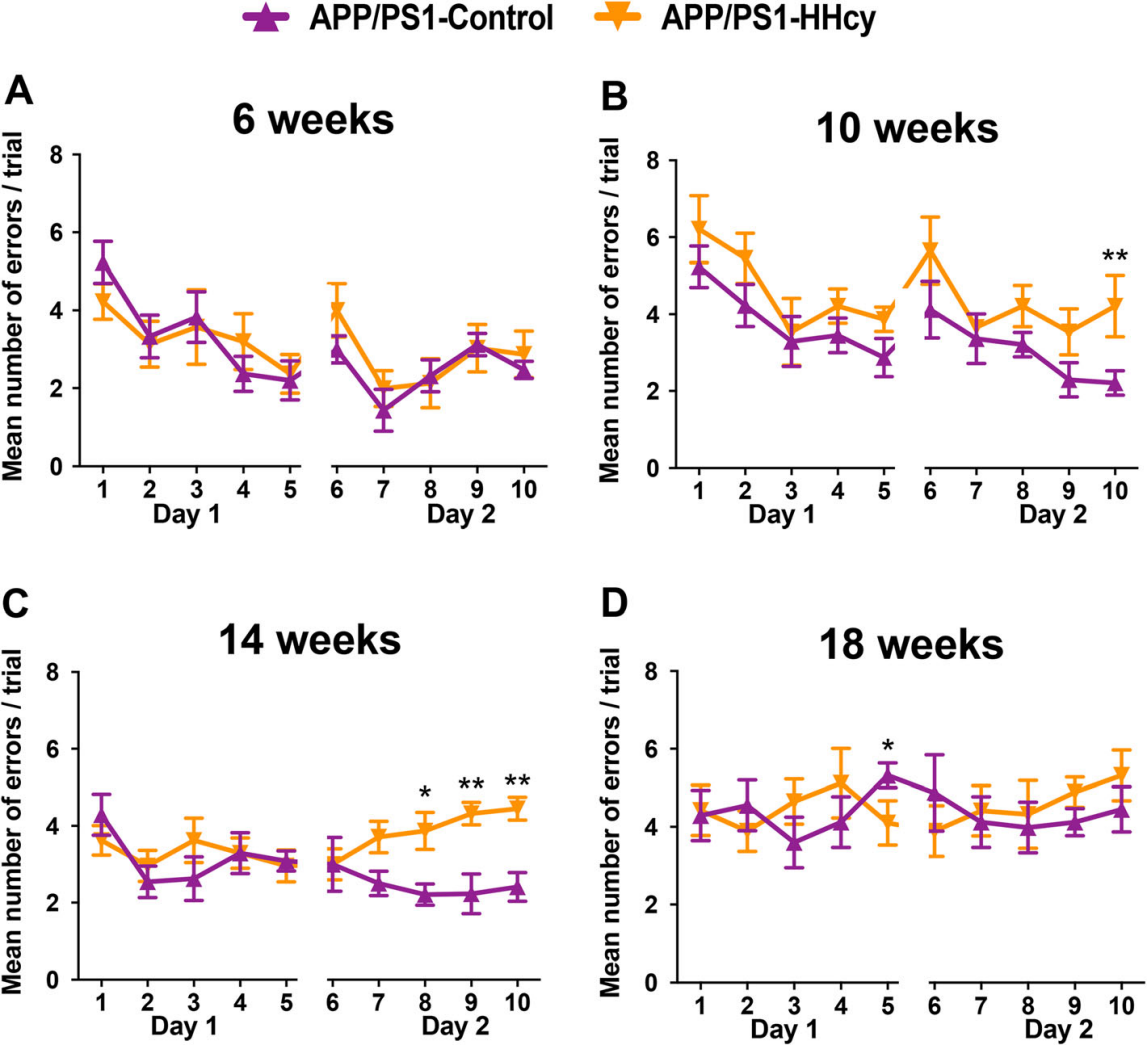
Spatial memory deficits are seen starting at 10 weeks on the HHcy diet in APP/PS1 mice. **a–d** Two-day radial arm water maze data is shown for APP/PS1 mice on diet for 6, 10, 14, or 18 weeks. The mean number of errors per trial was calculated for each block (each block is the average of three trials). * indicates *P* < 0.05 and ** indicates *P* < 0.01 by repeated measures ANOVA

We tested spatial memory changes in the APP/PS1 mice on the HHcy and control diets using the 2-day radial arm water maze. After 6 weeks on the HHcy diet, the APP/PS1 mice were not significantly cognitively impaired compared to the APP/PS1 mice on control diet (Fig. 3a). By 10 weeks on diet, the APP/PS1 mice on the HHcy diet made significantly more errors only in the last block of trials on the second day (Fig. 3b), similar to WT mice on the HHcy diet for 10 weeks [17]. The APP/PS1 mice on the HHcy diet made significantly more errors on the last three blocks on the second day at 14 weeks compared to the APP/PS1 mice on control diet (Fig. 3c). After 18 weeks on either the HHcy or the control diet, both groups of mice made a similar number of errors and did not learn the task at all (Fig. 3d). This does not mean that one group was more impaired than the other; the mice have reached a ceiling effect with this particular behavior task and further cognitive decline cannot be seen with this task. However, neither group showed any learning and are therefore still considered impaired.

**Fig. 4:**
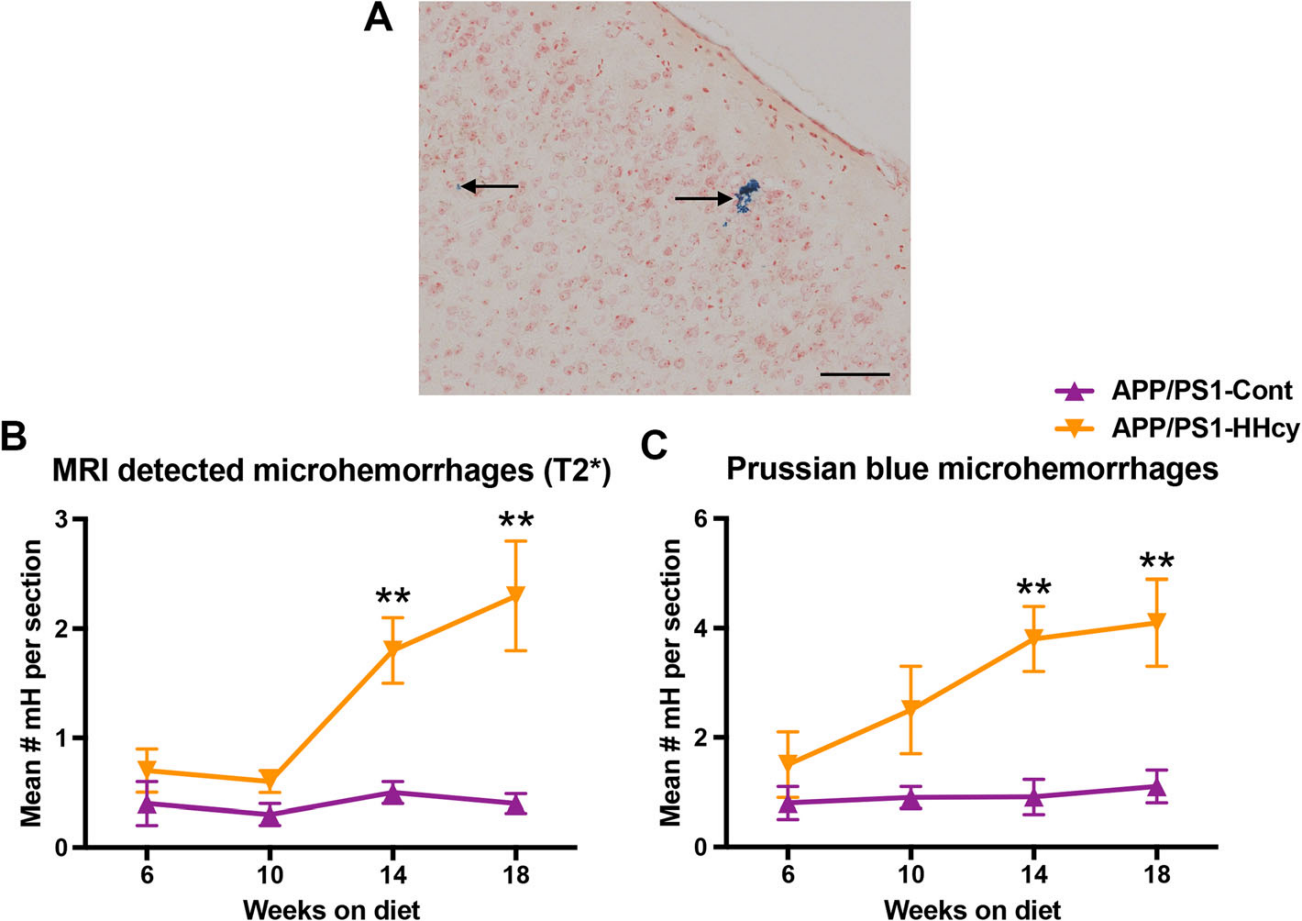
Microhemorrhages increase at 14 weeks on the HHcy diet in APP/PS1 mice. **a** Representative image of Prussian blue-positive microhemorrhage in the frontal cortex. Magnification = × 200. Scale bar = 50 μm, arrows indicate microhemorrhages. Quantification of the mean number of microhemorrhages per section from T2* MRI (**b**) or Prussian blue histology (**c**). ** indicates *P* < 0.01 compared to APP/PS1 mice on control diet for that time point

Using both T2* MRI and Prussian blue staining, we determined the time course of microhemorrhages due to the HHcy diet in our co-morbidity model. Both methods showed a significant increase in the number of microhemorrhages in the APP/PS1 mice beginning at 14 weeks on the HHcy diet compared to the APP/PS1 mice on the control diet (Fig. 4a–c). Again, in the WT mice on the HHcy diet, we see a significant increase in microhemorrhages at 14 weeks [17].

**Fig. 5:**
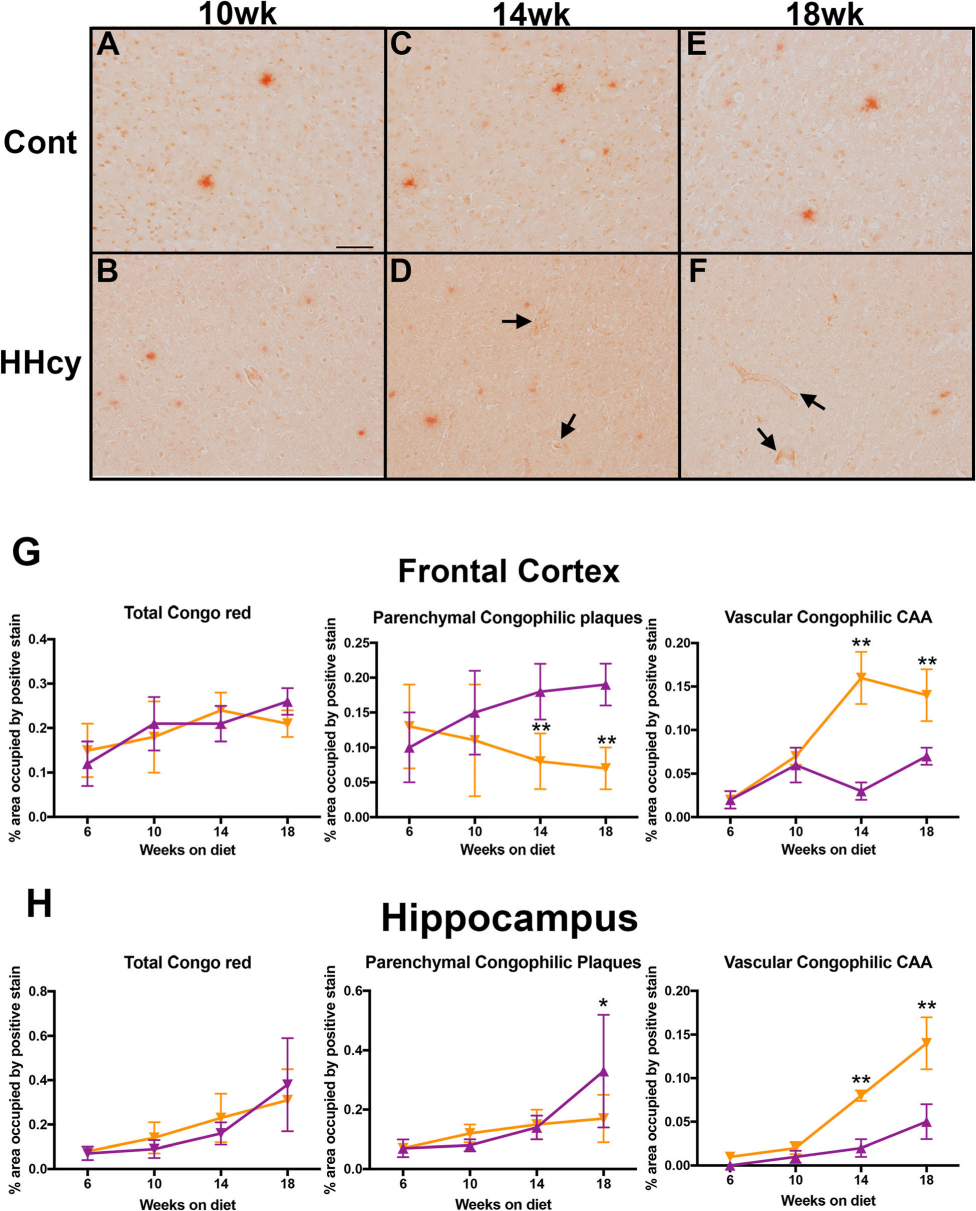
HHcy redistributes amyloid to the vasculature at 14 weeks on diet in the APP/PS1 mice despite no effect of HHcy on total Abeta levels. **a–f** Representative images of Congo red staining in the frontal cortex of APP/PS1 mice on control diet or HHcy diet for 10, 14, or 18 weeks are shown. Magnification = × 200X. Scale bar in *A* = 50 μm for all images. Black arrows indicate amyloid around a vessel. **g**-**h** Quantification of percent positive stain in the frontal cortex (**g**) and hippocampus (**h**). * indicates *P* < 0.05, ** indicates *P* < 0.01 compared to APP/PS1 mice on control diet at that time point

To determine brain beta-amyloid (Aβ) load, we performed biochemical assessment of soluble and insoluble Aβ_1–38_, Aβ_1–40_, and Aβ_1–42_ in the Meso-Scale Discoveries V-Plex Aβ assay. We found no significant effect of HHcy induction in Aβ levels at any of the time points examined (Table 3). To determine changes in amyloid distribution, we performed Congo red staining, which enables differentiation of vascular and parenchymal amyloid. At each time point, HHcy did not alter total levels of Congo red staining in both the frontal cortex and hippocampus (Fig. 5g, h). However, when parenchymal plaques and vascular amyloid are separated from total Congo red staining, it is revealed that HHcy leads to a redistribution of amyloid towards the vasculature (Fig. 5g, h). This redistribution occurs at 14 weeks on diet and continues to 18 weeks on diet (Fig. 5c–f).

**Table 3:** Biochemical Aβ assessment

Group	Time-point (weeks)	Aβ_1–38_		Aβ_1–40_		Aβ_1–42_	
		Soluble	Insoluble	Soluble	Insoluble	Soluble	Insoluble
Control	6	0.03 ± 0.01	12.2 ± 2.8	0.9 ± 0.2	410 ± 95.2	0.7 ± 0.2	619 ± 87.2
	10	0.05 ± 0.03	20.6 4.4	0.4 ± 0.1	550 ± 87.6	0.8 ± 0.3	670 ± 69.5
	14	0.08 ± 0.02	24.8 ± 6.8	0.8 ± 0.6	668 ± 100.1	1.4 ± 0.6	1214 ± 85
	18	0.12 ± 0.06	19.9 ± 8.7	1.5 ± 0.4	942 ± 206	1.2 0.4	1513 ± 97
HHcy	6	0.02 ± 0.01	13.2 ± 1.3	1.1 ± 0.3	570 ± 120.2	0.9 ± 0.3	555 ± 98.7
	10	0.14 ± 0.07	29.6 ± 9.4	0.8 ± 0.2	654 ± 126.4	0.7 ± 0.2	741 ± 59.7
	14	0.09 ± 0.03	22.5 ± 7.6	0.7 ± 0.1	781 ± 154.2	1.4 ± 0.6	1356 ± 96
	18	0.16 ± 0.08	19.6 ± 8.4	0.6 ± 0.2	1150 ± 215	1.5 ± 0.4	1641 ± 79

## Discussion

VCID is the second most common form of dementia yet it is commonly seen as a co-morbidity with AD [25–28]. While VCID is being increasingly recognized in the clinic, there are still a limited number of animal models to study mechanisms of VCID alone and when it is co-morbid with AD. Previously, we characterized a mouse model of VCID and a co-morbidity model by inducing HHcy in WT and APP/PS1 mice, respectively [16–18]. We have shown the time course of neuropathological events in our VCID model alone [17]; however, we do not know the effect of amyloid deposition on this time course. Understanding and comparing the time course of these pathological changes could provide critical information for the mechanism of VCID alone and when it is co-morbid with AD. In this study, we show that neuroinflammation is the first pathology to occur, followed by cognitive changes and finally microhemorrhages and amyloid redistribution.

While little is known about inflammation’s role in VCID, it is hypothesized that inflammation and oxidative stress play a key role in neurovascular dysfunction leading to cognitive decline. Chronic hypertension, a risk factor for VCID, leads to lumen narrowing and ultimately reduced blood flow. This decrease in blood flow induces expression of hypoxia inducible factor 1α which recruits macrophages from the systemic circulation as well as activation of endogenous microglia [29]. Activation of microglia leads to increases in proteases and free radicals that are released during remodeling of damaged vessels. Free radicals and proteases can break down the fibrotic basal lamina, disrupt tight junctions leading to vasogenic edema, and attack the myelinated fibers leading to demyelination [30, 31]. This increase in reactive oxygen species can also stimulate inflammatory pathways via toll-like receptors and lead to blood brain barrier breakdown. This vascular damage via inflammation most likely interferes with neurovascular coupling and the proliferation, migration, and differentiation of oligodendrocytes contributing to white matter damage and VCID [32]. In both our VCID and co-morbidity model, alterations in the inflammatory phenotype are the first pathology to appear along with an increase in microglial staining. In addition, both models show a significant increase in several pro-inflammatory markers starting at 6 weeks before any disruptive vessel changes or cognitive decline is seen. While the microglial staining returned to control levels by 18 weeks on diet, the increase in pro-inflammatory markers remains throughout the course of the diet, possibly continuing to contribute to the neuropathological changes seen later. Future studies will look more closely at the morphological phenotype of the microglia to determine the number of activated vs inactive microglia. Together, this suggests neuroinflammation is an initial, key player in homocysteine-induced VCID.

As mentioned above, the proteases and free radicals released by activated microglia can induce blood brain barrier breakdown and leakage. Matrix metalloproteinase 9 (MMP9) is a key protease shown to be activated by pro-inflammatory cytokines (IL-1β and TNFα) and has been shown to degrade tight junction proteins leading to leaky vessels and eventually microhemorrhages [33–35]. We have previously shown a significant increase in MMP9 gene expression and protease activity in both our VCID and co-morbidity models, with gene expression of MMP9 being significantly elevated after 24 weeks on diet [16, 18, 36]. MMP9 gene expression is still significantly elevated in the co-morbidity mice after 24 weeks on diet, showing a continued expression even after inflammation is returned to control levels [37]. In the current study, we show a slight increase in microhemorrhages at 10 weeks, with a significant increase by 14 weeks on diet. The appearance in microhemorrhages also coincides with the cognitive changes as well in both our VCID and co-morbidity models. At 10 weeks, there is a slight decline in cognition that becomes more pronounced by 14 weeks. With neuroinflammatory changes occurring before the appearance of microhemorrhages, we hypothesize that neuroinflammation induces MMP9 activation leading to microhemorrhages and then cognitive decline.

Another target of MMP9 includes the dystroglycans, specifically β-dystroglycan, which forms a complex that anchors the astrocytic end-foot to the basement membrane surrounding arterioles and capillaries [38, 39]. As part of the neurovascular unit, astrocytic end-feet and their anchoring complexes function to maintain the ionic and osmotic homeostasis of the brain. Disruption of these homeostatic processes by breakdown of the anchoring complex and removal of the end-feet from the vasculature could lead to cognitive decline. Previously, we have shown a significant decrease in several astrocytic end-feet markers by 10 weeks on diet in our WT mice [17]. This coincides with the slight increase in microhemorrhages at 10 weeks as well as the slight behavioral deficits at 10 weeks seen in both our models. In addition, it has been shown that astrocytic end-foot disruption is associated with cerebral amyloid angiopathy in both AD and mouse models of cerebral amyloid angiopathy [20]. In our co-morbidity model, since we have shown a redistribution of Congophilic amyloid from the parenchyma to the vasculature that occurs at 14 weeks on diet, we hypothesize that, similar to the WT HHcy mice, the co-morbidity mice will also have astrocyte end-feet degeneration around a similar time point. If so, with microhemorrhages and astrocytic end-foot disruption occurring before the redistribution of amyloid, astrocytes could play a key role in clearing amyloid through the vasculature. Any disruptions in the astrocytes could then lead to hindered clearance and thus the redistribution seen in our co-morbidity model.

## Conclusions

Taken together, we have shown that neuroinflammation, specifically, an elevation of pro-inflammatory cytokines, is the initial change in both our VCID and co-morbidity mouse models and that the presence of amyloid does not alter the time course of neuropathological events in our mouse model. Overall, this data provides several therapeutic targets and time points that can be tested in our mouse models as well as potential overlapping pathologies to look for in other VCID and AD models and human tissue.

## Data Availability

Data sharing is not applicable to this article as no datasets were generated or analyzed during the current study.

## Abbreviations

AD: Alzheimer’s disease
Hcy: Homocysteine
HHcy: Hyperhomocysteinemia
IL10: Interleukin 10
IL12a: Interleukin 12 a
IL1β: Interleukin 1 beta
IL-6: Interleukin 6
MMP: Matrix metalloproteinase
TNFα: Tumor necrosis factor alpha
VCID: Vascular contributions to cognitive impairment and dementia
WT: Wild-type
YM1: Chitinase-like 3
